# Heavy Metal Levels in Males With Idiopathic Hypogonadotropic Hypogonadism

**DOI:** 10.7759/cureus.53128

**Published:** 2024-01-28

**Authors:** Serpil Ciftel, Alev Lazoglu Ozkaya

**Affiliations:** 1 Department of Endocrinology and Metabolism, Erzurum Health Science University, Erzurum, TUR; 2 Department of Biochemistry, Erzurum Health Science University, Erzurum, TUR

**Keywords:** cadmium, arsenic (as), testosterone, heavy metal, idiopathic hypogonadotropic hypogonadism

## Abstract

Introduction: The toxic effects of heavy metals on biological systems are being investigated with increasing interest day by day. Our purpose was to investigate heavy metals such as aluminum (Al), cadmium (Cd), arsenic (As), lead (Pb), and nickel (Ni) in males with idiopathic hypogonadotropic hypogonadism (IHH) and to determine whether there is a relationship between heavy metals and testosterone levels.

Methods: Twenty-six male patients with IHH aged 18-50 and 22 healthy males aged 21-50 admitted to the Outpatient Department of Endocrinology for follow-up were enrolled. BMIs were calculated by measuring the height and weight of all participants. Al, Cd, As, Pb, and Ni levels were measured and compared between groups. Testosterone levels were measured to investigate whether there was a correlation with heavy metal levels.

Results: Al, Cd, As, Pb, and Ni levels were statistically higher in the patient group compared to the control group (p<0.001). A moderately strong significant negative correlation was detected between the patients' testosterone and As levels (p=0.001, r=-0.609, R^2^=0.371). Decreased As and Cd levels were observed as the patients’ ages increased (p=0.013, r=-0.471).

Conclusion: Heavy metals might play potential roles in IHH. We hope that investigating heavy metal levels in IHH and adding toxicity-preventive treatments to hormonal therapies will be beneficial in the multifaceted management of the disease in clinical practice.

## Introduction

Isolated hypogonadotropic hypogonadism (IHH) is an endocrine disorder that can be congenital or acquired, resulting from the absence or decrease in gonadotropin hormone-releasing hormone (GnRH) secretion from the hypothalamus or follicle-stimulating hormone (FSH) and luteinizing hormone (LH) secretion from the pituitary gland, and other anterior pituitary hormones are detected normal levels. The etiology of congenital isolated hypogonadotropic hypogonadism includes syndromic causes and gene mutations. To date, identified genes explain only about 50% of the cases. Therefore, unidentified genes are also strongly considered possible [1]. In the etiology of acquired IHH, use of drugs such as opiates and glucocorticoids, alcohol abuse, severe or chronic illness, infiltrative or infectious pituitary lesions, radiation and hemochromatosis, sarcoidosis and histiocytosis X, serious diseases (e.g. cancer, AIDS, and starvation) or chronic diseases (e.g. end-stage renal and liver disease and morbid obesity) are blamed [2]. It is known that one of the causes of IHH is excessive iron overload due to juvenile hemochromatosis. Unlike these, there are also idiopathic cases in which no underlying cause can be identified. IHH can be detected with increasing frequency, even in men with typical sexual characteristics and who have children. Thus, the importance of triggering factors is growing day by day. However, studies on the significance of heavy metals in IHH have not been conducted yet.

The growing interest in the harmful effects of heavy metals in recent years is opening the door to a new research perspective on how these toxic compounds might affect the endocrine system. Heavy metals are elements that can have harmful effects even at low concentrations. As technology continues to advance, heavy metals in drinking water, the most common cause of exposure, are exceeding upper reference limits worldwide. Due to the widespread use of modern industrial agriculture, mining, metallurgy, processed food, and transportation vehicles, even trace amounts of exposure through skin contact, oral, and inhalation cause accumulation in the body due to their slow metabolic excretion [3]. Acute and toxic dose exposures are emergencies that require immediate diagnosis and treatment. However, the effects of low/moderate level chronic exposure are less known as they might be subclinical and manifest clinically over time with various presentations. The primary causes of the toxic effect are intracellular metabolic processes, increased oxidative stress and oxidative protein degradation, mitochondria damage, and induction of apoptosis. The endocrine system is susceptible to the harmful effects of heavy metals. Exposure to endocrine-disrupting chemicals, including heavy metals, may negatively affect hormone synthesis, metabolism, and transport. Even exposure in utero may lead to endocrine problems that might persist long after birth [4]. Heavy metals such as cadmium (Cd), arsenic (As), lead (Pb), aluminum (Al), and nickel (Ni) are among the most well-known. Cd [5] and As [6] can disrupt the repair function of DNA damage, leading to genetic mutations and various cancers. Exposure to As may alter the hypothalamic-pituitary-gonadal (HPG) axis, decreasing testosterone synthesis and spermatogenesis [7]. As a nutritional antioxidant, zinc is crucial in maintaining various cellular pathways. It has been reported that zinc deficiency in men reduces testosterone levels, and zinc supplementation benefits biological processes like androgen balancing [8]. Additionally, zinc supplementation is beneficial in chronic As exposure [9]. Pb exposure during the fetal period negatively affects reproductive potential by disrupting hormonal synthesis in both genders [10]. Exposure to Ni, a common ecological pollutant, has been shown to cause inhibition of the expression of critical genes in the hypothalamic pituitary testicular axis and testicular and epididymal histopathological lesions in mice studies [11]. Al, the most common metal in our environment, can negatively affect testosterone synthesis, testicular histomorphometry, and sperm parameters at high and low levels of exposure [12]. 

It is significant to investigate the harmful effects of heavy metals, including aluminum, arsenic, cadmium, nickel, and lead, on the reproductive system to gain a comprehensive understanding of hormonal problems. How these heavy metals can affect the endocrine system, especially the HPG axis, may be important in elucidating the possıble relationships with IHH. There is no previous study investigating the levels of heavy metals in IHH.

Our study aims to investigate serum heavy metal levels in males with IHH, compare them to healthy ones, and contribute to understanding whether there is potential heavy metal exposure in these patients. In this way, this study can provide a significant scientific basis for developing disease prevention strategies and adding treatments for heavy metal toxicity in addition to hormonal replacement therapies in IHH.

## Materials and methods

Study population

The diagnostic criteria employed for isolated idiopathic hypogonadotropic hypogonadism in males included the following: (a) the absence of pubertal development by the age of 18, (b) serum testosterone level ≤ 100 ng/dl with low serum gonadotropin levels in the first visit; (c) other anterior pituitary hormone levels remaining normal; and (d) no abnormalities in hypothalamic-pituitary region magnetic resonance imaging. This cross-sectional study enrolled 26 male patients with a mean age (standard deviation) of 28.1 (8.4) with IHH and 22 healthy males with a mean age (standard deviation) of 31.5 (9.8) who visited the Endocrinology Outpatient Clinic of Erzurum Training and Research Hospital between May 2023 and December 2023. No genetic mutation was found in the genetic analysis performed on patients with isolated hypogonadotropic hypogonadism. Patients below 18 years, female gender, IHH with a known etiology, and patients receiving hormonal treatments other than androgen replacement were excluded from the study. The patient group was receiving either testosterone preparation (n=8) or chorionic gonadotropin alpha (n=18) treatments. There were no differences in the patient and control groups' mean ages, BMIs, and smoking rates.

The study protocol received approval from the Ethics Committee of Erzurum Training and Research Hospital, with a decision number 2023/01-13, dated 31.05.2023. All participants provided written and verbal informed consent. The research was conducted in accordance with the ethical guidelines outlined in the Declaration of Helsinki. 

Laboratory data

Serum samples obtained from the participants were taken into empty biochemistry tubes between 08.00 and 09.00 in the morning and centrifuged for 10 minutes at 4000 rpm. Total testosterone (TT), FSH, and LH levels were analyzed using the chemiluminescence immunoassay method in the Siemens Atellica IM device (Munich, Germany). Al, Cd, As, Pb, and Ni levels were measured by ICAP RQ inductively coupled plasma mass spectrometry (ICP-MS) (Thermo Scientific, Waltham, MA, USA). Hormones and heavy metals were analysed in simultaneously taken blood samples. Weight and height were measured, and body mass index (BMI) was calculated by using the formula of weight in kg/m^2^.

Statistical analysis

Statistical Package for Social Sciences (SPSS) version 22.0 (IBM Corp., Armonk, NY, USA) statistical software was used for all analyses. Before the analyses, normality tests were performed using Shapiro-Wilk and skewness-kurtosis tests. The Student's t-test and the Mann-Whitney U Test were used to compare normally and non-normally distributed variables in comparisons of two independent groups. Students' t-tests were used to compare the mean values of the heavy metals and TT levels in the patient group. Spearman's correlation analysis was used in the patient group to evaluate the correlation between TT and heavy metals. The Mann-Whitney U test was used to compare the patient and control groups. The chi-square test was employed for 2x2 comparisons. Linear regression analysis was used to identify the relationship between arsenic and testosterone levels. All significant values were defined by p<0.05.

## Results

There were no differences in the mean ages, BMIs, and smoking rates of the patient and control groups (p=0.188, p=0.375, p=0.594, respectively). The mean age was 28.1±8.4 years, and the mean level of TT was 344±286 ng/dl in the patient group. Serum FSH, LH, and testosterone levels were significantly lower in the patient group. The concentrations of Al, Cd, As, Pb, and Ni were significantly different between the two groups. All these heavy metals were significantly higher in the patient group than in the control group (p<0.001) (Table 1).

**Table 1:** Demographic characteristics and heavy metal levels of study and control groups Not normally distributed data were expressed as medians (interquartile range). Age, BMI, and smoking rate are expressed as mean± standard deviation. Abbreviations: IHH: Idiopathic Hypogonadotropic Hypogonadism, BMI: Body Mass Index, FSH: Follicle stimulating hormone, LH: Luteinizing hormone

	Patients with IHH (n= 26)	Control group (n=22)	p
Age (year)	28.1±8.4	31.5±9.8	0.188
BMI (kg/m^2^)	23.8±2.6	24.5±2.36	0.374
Smoking rate n(%)	11 (42.3)	11 (50.0)	0.594
Testosterone (ng/dl)	344±286	549±147	0.003
FSH (mIU/mL)	0.60 (0.01-1.77)	4.54 (2.30-6.50)	<0.001
LH (mIU/mL)	0.21 (0.00-1.20)	3.92 (2.34-6.20)	<0.001
Aluminum (μg/L)	0.534 (0.406-0.970)	0.145 (0.139-0.166)	<0.001
Cadmium (μg/L)	3,571 (2.04-5.55)	2.173 (1.923-2.777)	<0.001
Arsenic (μg/L)	0.077 (0.057-0.0910)	0.043 (0.039-0.059)	<0.001
Lead (μg/L)	5.025 (4.06-9.43)	2.444 (2.392-2.500)	<0.001
Nickel (μg/L)	1.960 (1.72-2.17)	1.219 (1.136-1.369)	<0.001

A moderately strong significant negative correlation was detected between the patients' testosterone levels and arsenic (p=0.001, r=-0.609, R^2^=0,371). No significant correlation was detected between other heavy metals such as aluminum, cadmium, lead, nickel, and testosterone (p=0.905, p=0.881, p=0.750, p=0.588, respectively). Additionally, As and Cd levels decreased as the patients’ ages increased (r=-0.471, p=0.013; r=-0.419, p=0.037, respectively) (Table 2).

**Table 2:** Spearman correlation analysis of heavy metals, testosterone, and age in the patient group

	Testosterone		Age	
	r	p	r	p
Aluminium	0.025	0.905	-0.200	0.327
Cadmium	0.031	0.881	-0.419	0.037*
Arsenic	-0.609	0.001*	-0.602	0.001*
Lead	0.067	0.750	0.190	0.362
Nickel	-0.116	0.588	0.107	0.618

According to linear regression analysis model results regarding the effect of As on testosterone levels in the patient group, As concentration was statistically effective on the testosterone level (p=0.001, R^2^=0.371, β=-0.609) (Table 3).

**Table 3:** Simple linear regression analysis between arsenic and testosterone levels R=0.609   R^2^=0.371 F=14.7 p=0.001

Variable	B	SE	β	t	p
-	1530	307	-	4.975	<0.001
Arsenic	-15352	3997	-0.609	-3.840	0.001

## Discussion

IHH is an endocrine disorder characterized by the impairment of the HPG axis, whose etiology hasn’t yet been fully elucidated. The focus of this study is to investigate the significance of heavy metals, including Al, Cd, As, Pb, and Ni, considering the lack of literature on the roles of heavy metals in IHH. Heavy metals are known as endocrine disruptors based on ongoing current research. Most studies exploring the relationship between the hypothalamic-pituitary-testis axis and heavy metals focus on the impact on testosterone levels in healthy males without hypogonadism [13]. Unlike all these other studies, our study was conducted in males with IHH in addition to healthy males. To the best of our knowledge, no studies have shown the relationship between IHH and heavy metals. The results of our study demonstrated higher heavy metal concentration in males with IHH compared to healthy men. Furthermore, decreased serum testosterone is significantly associated with a high level of As. Hsieh et al. also demonstrated that chronic As exposure lowers testosterone levels, which is consistent with our study [14]. A study conducted on late-onset hypogonadism in males over the age of 50 demonstrated a negative correlation between Cd and free testosterone and no correlation between As and testosterone levels, in contrast to our study [15]. The inconsistency of findings in studies might be attributable to the variation of age ranges and the heterogeneity of underlying diseases. In particular, our detection of a negative relationship between serum As level and testosterone level further strengthens the role of this metal in the pathogenesis of hypogonadism. Arsenic may impair the function of Leydig cells by increasing oxidative stress in testicular tissue. Additionally, arsenic may affect testosterone levels by inhibiting gonadotropin secretion in the hypothalamus or pituitary gland. In one of the mice studies, male offspring with paternal arsenic exposure were exposed to arsenic until puberty, which led to detrimental effects on HPG axis functionality through mechanisms of oxidative stress, autophagy, and mitochondrial impairment [16].

In our current study, the decrease in serum As and Cd levels with increasing age may indicate that these metals' metabolism and detoxification mechanisms could alter with age. This phenomenon could be attributed to the deposition of heavy metals in bones rather than in the bloodstream [17].

One of the limitations of our study is that it was conducted in a single center with a limited number of patients. However, since it is a rare disease, the patient count is considered to be expected for a single-center cross-sectional study. So, it can not prove causality. More studies need to be conducted considering all etiological factors at the time of new diagnosis to prove the causality of this relationship. Another limitation due to the rarity of the idiopathic type is that testosterone levels are measured in patients receiving treatment, not in newly diagnosed patients. In addition, including only male subjects is one of the reasons for the limited number of patients. However, including only male individuals with a non-elderly age range is one of the study's strengths regarding the accuracy and generalizability of the results for males with IHH.

The molecular mechanisms of male reproductive toxicity caused by heavy metals remain unclear but continue to be investigated [18]. Although inflammatory response, oxidative stress, autophagy, and apoptosis are potential mechanisms, multicentric studies with an adequate number of patients are needed to investigate the molecular mechanisms in more detail to elucidate the causal relationship between heavy metals and the pathogenesis of IHH. Trying to reveal the underlying causes of IHH, which impairs the quality of life in terms of physical, psychological, and fertility concerns from the adolescence period onward, is of great importance. Our study will make a significant contribution to the literature in terms of discovering treatments for heavy metal toxicity, such as antioxidants, in addition to hormonal replacement therapies, with a multidisciplinary approach to prevent these factors or reduce their harmful effects.

## Conclusions

The relationship between heavy metal exposure and IHH is important for understanding the toxic effects on the endocrine system. This study shows elevated heavy metal levels in patients with IHH, suggesting that environmental factors may play a potential role in the development of this endocrine disorder. The present study will pave the path for mechanism-specific antioxidant treatments to reduce and reverse the harmful effects of heavy metal exposure in IHH. All these findings suggest raising awareness about investigating heavy metals in IHH and developing treatment and disease prevention strategies in the future. Also, governments should implement stringent measures to reduce heavy metal levels in drinking and groundwater, food, and industrial wastewater, which are essential ways of transmission, thus reducing the exposure levels of humans and animals.
